# Subnitrate Bismuth—Adulteration

**Published:** 1875-01

**Authors:** 


Adulterated Bism. Subnit.—The patient was suffering from an extensive burn of the leg. Bism. subnit. was dusted thickly over the open surface and covered with dry lint. In a few days symptoms of metallic poisoning exhibited themselves; the patient complaining of increased pain at the seat of the injury; a metallic taste in the mouth; a blue line being plainly visible upon the gums, and the cheeks were the seat of numerous small ulcers. The bismuth was tested, and exhibited the presence of lead in large quantities. The dressing was immediately discontinued, and marked improvement in the condition of the patient followed the substitution of a dressing of simple cerate and the internal administration of potas. iodid. in doses of grs. x. three times daily.—*Medical Journal.*

